# Expression profiling of genes involved in drought stress and leaf senescence in juvenile barley

**DOI:** 10.1186/s12870-015-0701-4

**Published:** 2016-01-05

**Authors:** Gwendolin Wehner, Christiane Balko, Klaus Humbeck, Eva Zyprian, Frank Ordon

**Affiliations:** Julius Kühn-Institut (JKI), Federal Research Centre for Cultivated Plants, Institute for Resistance Research and Stress Tolerance, Rudolf-Schick-Platz 3, 18190 Sanitz, Germany; Interdisciplinary Center for Crop Plant Research (IZN), Hoher Weg 8, 06120 Halle, Germany; Martin-Luther-University Halle-Wittenberg, Institute of Biology, Weinbergweg 10, 06120 Halle, Germany; Julius Kühn-Institut (JKI), Federal Research Centre for Cultivated Plants, Institute for Grapevine Breeding, Geilweilerhof, 76833 Siebeldingen, Germany; Julius Kühn-Institut (JKI), Federal Research Centre for Cultivated Plants, Institute for Resistance Research and Stress Tolerance, Erwin-Baur-Str. 27, 06484 Quedlinburg, Germany

**Keywords:** Barley, Leaf senescence, Drought stress, High-throughput qPCR, Gene expression, eQTL

## Abstract

**Background:**

Drought stress in juvenile stages of crop development and premature leaf senescence induced by drought stress have an impact on biomass production and yield formation of barley (*Hordeum vulgare* L.). Therefore, in order to get information of regulatory processes involved in the adaptation to drought stress and leaf senescence expression analyses of candidate genes were conducted on a set of 156 barley genotypes in early developmental stages, and expression quantitative trait loci (eQTL) were identified by a genome wide association study.

**Results:**

Significant effects of genotype and treatment were detected for leaf colour measured at BBCH 25 as an indicator of leaf senescence and for the expression level of the genes analysed. Furthermore, significant correlations were detected within the group of genes involved in drought stress (r = 0.84) and those acting in leaf senescence (r = 0.64), as well as between leaf senescence genes and the leaf colour (r = 0.34). Based on these expression data and 3,212 polymorphic single nucleotide polymorphisms (SNP) with a minor allele frequency >5 % derived from the Illumina 9 k iSelect SNP Chip, eight *cis* eQTL and seven *trans* eQTL were found. Out of these an eQTL located on chromosome 3H at 142.1 cM is of special interest harbouring two drought stress genes (GAD3 and P5CS2) and one leaf senescence gene (Contig7437), as well as an eQTL on chromosome 5H at 44.5 cM in which two genes (TRIUR3 and AVP1) were identified to be associated to drought stress tolerance in a previous study.

**Conclusion:**

With respect to the expression of genes involved in drought stress and early leaf senescence, genotypic differences exist in barley. Major eQTL for the expression of these genes are located on barley chromosome 3H and 5H. Respective markers may be used in future barley breeding programmes for improving tolerance to drought stress and leaf senescence.

**Electronic supplementary material:**

The online version of this article (doi:10.1186/s12870-015-0701-4) contains supplementary material, which is available to authorized users.

## Background

In order to analyse genetic networks and stress response, real time polymerase chain reaction (PCR) is an important tool [1]. For several years high-throughput instruments e.g. the BioMark System from Fluidigm have enabled large scale quantitative PCR studies [2]. Because of this and the possibility to analyse a large number of genotypes easily on expression chips [2] a range of genome wide association studies (GWAS) using expression data were conducted in the last years [3–5]. Expression quantitative trait loci (eQTL) were detected first in medicinal studies in humans and later also in plants [6–10]. In plants most eQTL studies were performed for complex pathways and aimed at a better understanding of the molecular networks [11]. Whereas in biotic stress the resistance is often controlled by a single gene, responses to abiotic stresses such as drought stress are controlled by many genes [12–14] and so these processes are particularly suitable for high throughput expression analyses and genetical genomics approaches [15]. Even in early developmental stages drought stress and drought stress induced premature leaf senescence have major influences on yield formation [16]. Therefore, it is of prime importance to understand regulatory processes of drought stress [17] and leaf senescence [18].

In plants drought stress is initiated by water deficit in soil resulting in osmotic and oxidative stress and cellular damage [19]. This leads to defined drought stress responses for instance regarding the maintenance of turgor by an increase of osmoprotective molecules as soluble sugars [20–22], as well as measurable lower water content and decreased growth in the stressed plants compared to a control [23, 24]. Stress perception is assigned by special receptors, such as abscisic acid (ABA) receptors, hexokinases, or ion channel linked receptors [25]. The stress signal is then transducted for example via serine-threonine kinases, serin-threonine phosphatases, calcium dependent protein kinases, or phospholipases [25]. Finally, the gene expression is regulated by effector genes coding for late embryo abundant (LEA) proteins, dehydrin, or reactive oxygen species (ROS) and transcription factors, such as MYB, WRKY, NAC, AP2/ERF, DREB2, or bZIP to activate stress responsive mechanisms, re-establish homeostasis and protect and repair damaged proteins and membranes [13, 19, 25, 26]. Besides the above mentioned genes, drought stress associated metabolites such as osmoprotectants, polyamines and proteins involved in carbon metabolism and apoptosis are part of drought stress tolerance [12, 27]. Disturbing the regulatory processes in drought stress response results in irreversible changes of cellular homeostasis and the destruction of functional and structural proteins and membranes, leading to cell death [19] and decreased yield formation [28]. A huge transcriptome analysis for drought stress associated genes was done for example in barley [29] and wheat [30] showing differential response of genes involved in drought stress tolerance.

Initiated by external signals e.g. various stresses such as drought, as well as by internal factors for example phytohormones leaf senescence often occurs as a natural degradation process at the final stage of plant development [31]. Drought stress induced leaf senescence proceeds in three steps. Perception of drought stress is the initiation phase in which senescence signals are transferred via senescence associated genes (SAG) [32]. These are regulatory genes which often encode transcription factors regulating gene expression by binding to distinct *cis*-elements of target genes [33]. In the following reorganisation phase resources are transported from source (e.g. roots, leaves) to sink (e.g. fruits, seed) organs being important for yield formation [34]. With this translocation chlorophyll, proteins, lipids and other macromolecules are degraded and the content of antioxidants, ABA and ROS increases induced by a change in gene expression [35, 36]. Differentially expressed genes and their regulation during leaf senescence were identified by transcriptome analysis using microarrays in *Arabidopsis thaliana* [37, 38]. While the genes for photosynthesis and chloroplast development are down-regulated, the genes for the degradation of macromolecules and recycling of resources are up-regulated [39]. For example, expressed genes for chlorophyll degradation are *PA42*, *Lhcb4* and *psbA* [40] and genes for N mobilization and transport are transcription factors WRKY [41] and NAC [42] as well as glutamine synthetase [38]. Genes differentially expressed can be grouped to those accelerating leaf senescence and genes delaying leaf senescence [43]. The latter possibly resulting in a “stay green” effect and improved drought tolerance [34, 44]. The reorganisation phase is the crucial step for reversibility, after which senescence is irreversible and leads to the final step where leaves and cells often die [45].

In barley (*Hordeum vulgare* L.), a crop plant of worldwide importance, most mechanisms for leaf senescence are still not well understood [18, 34]. The response to drought in juvenile stages is less well documented, as only few studies are focused on early developmental stages [20, 24, 46, 47] whereas a lot of studies were conducted for drought stress in the generative stage [48]. Nevertheless, barley is to some extent a model organism for research at a genome wide level. The barley gene space has been published [49] and with this information gene positions can be compared to these data. Comparing the position of the analysed genes in the Morex genome with positions of the detected eQTL, resulted in the co-localization of eQTL and genes involved in drought stress [11, 50]. Therefore, the present study aimed at the identification of eQTL in barley for genes involved in drought stress in the juvenile phase and early leaf senescence (Table 1) based on a genome wide association study.

**Table 1:** Primer pairs for the selected genes and the reference gene

	Gene	Functional annotation	Acc. No.	Primer (FOR and REV)	Ampl.
Drought stress genes	A1	ABA inducible gene	GenBank: X78205.1	ACACGGCGCAGTACACCAAGGAGT CCCACCACGGCGTTCACCAC	100 bp
	Dhn1	Dehydrin 1	GenBank: AF181451	GCAACAGATCAGCACACTTCCA GCTGACCCTGGTACTCCATTGT	141 bp
	GAD3	Glutamate decarboxylase 3	GenBank: AY187941	ATGGAGAACTGCCACGAGAA GGAGATCTCGAACTCGTCGT	147 bp
	NADP_ME	NADP-dependent malic enzyme-like	GenBank: XM_003569737	ATGGCGGGAAGATCAGGG ATCCCTCAGCAGGGAATGC	165 bp
	P5CS2	Delta 1-pyrroline-5-carboxylate synthase 2	GenBank: AK249154.1	GTATACATGCACGTGGACCC CAGAGGGTTTTCGCCGAATC	164 bp
Leaf senescence genes	Contig7437	SAG senescence associated gene	GenBank: KF190467.1	GCTGAACGGCTGCCACTCCC GAAACCATCGCGCCTGTGGTG	78 bp
	GSII	Glutamine synthetase 2	GenBank: X53580.1	ACGAGCGGAGGTTGACAG CGCCCCACACGAATAGAG	94 bp
	hv_36467	SAG senescence associated gene	GenBank: AK367894.1	CAGTCCTTTTGCGCAGTTTTC CCAAGCGAGAATGCCTTGTAA	152 bp
	LHC1b20	Light-harvesting complex I	GenBank: S68729.1	CTGACCAAGGCGGGGCTGATGAAC TCGTGGGGCGGGAGGCTGTAG	200 bp
	pHvNF-Y5α	SAG senescence associated gene	GenBank: AK370570	CATGAAGCGAGCTCGTGGAACA GGTGCGAAGGTGGGACTACTCTGA	126 bp
Genes out of GWAS^a^	AVP1	Vacuolar proton-inorganic pyrophosphatase	GenBank: AY255181.1	GACCCTCTCAAGGACACCTC TCCCAACCGGCAAAACTAGA	160 bp
	ETFQO	Electron transfer flavoprotein-ubiquinone oxidoreductase	GenBank: BT000373.1	CCACAACCCTTTCTTGAATCCG GATCTAAGGGCGTGGTGAATTT	160 bp
	SAPK9	Serine/threonine protein	GenBank: AB125310.1	TCATGCAAGACTGTTTCTTGGG TTTCTTCTTGGCACAAAGCATATT	149 bp
	TRIUR3	Protein kinase			
			GenBank: M94726	ACATTGACGTTGAGAGCAGC GCTACAGAGAATTTGTGACCCA	151 bp
	HvGAPDH	Glyceraldehyde-3-phosphate dehydrogenase	GenBank: DQ196027.1	CAATGCTAGCTGCACCACCAACTG CTAGCAGCCCTTCCACCTCTCCA	165 bp

^a^Genes coding for proteins identified by BlastX of significant marker sequences out of a previous genome wide association study (GWAS) by Wehner et al. [20]

## Results

### Leaf senescence

**Fig. 1:**
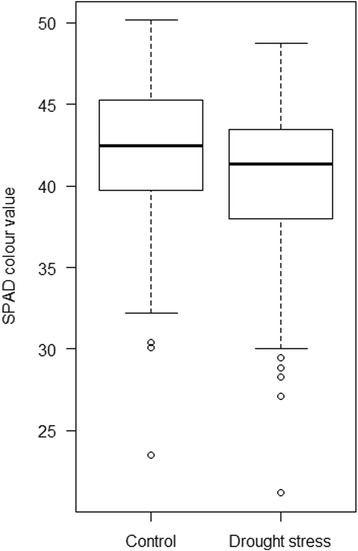
Box whisker plots for status of leaf senescence. Leaf colour (SPAD) for control and drought stress treatment at 27 days after sowing (das) including all 156 analysed barley genotypes

Leaf colour (SPAD, soil plant analysis development) measured at 20 days after drought stress induction (BBCH 25, according to Stauss [51]) being indicative for leaf senescence revealed significant differences between treatments and genotypes but no significant interaction of genotype and treatment was observed at this stage (Fig. 1 and Table 2) giving hint to physiological changes and changes in gene expression.

**Table 2:** Analysis of variance for leaf colour (SPAD) and the expression of the selected genes

	Trait/Gene	Effect of treatment		Effect of genotype	
		*F* value	*p* value	*F* value	*p* value
	SPAD	11.2	0.0009	6.6	<2E-16
Drought stress genes	A1	50.1	4.88E-12	8.8	<2E-16
	Dhn1	138.4	<2E-16	23.5	<2E-16
	GAD3	81.8	<2E-16	96.7	<2E-16
	NADP_ME	315.5	<2E-16	4.1	4.63E-09
	P5CS2	229.6	<2E-16	335.4	<2E-16
Leaf senescence genes	Contig7437	0.9	0.342	128.7	<2E-16
	GSII	175.4	<2E-16	65.1	<2E-16
	hv_36467	160.2	<2E-16	46.9	<2E-16
	LHC1b20	102.4	<2E-16	156.7	<2E-16
	pHvNF-Y5α	76.5	<2E-16	196.4	<2E-16
Genes out of GWAS^a^	AVP1	51.4	2.06E-12	37.9	<2E-16
	ETFQO	16.3	5.98E-05	41.3	<2E-16
	SAPK9	9.0	0.00312	5.8	2.88E-07
	TRIUR3	96.5	<2E-16	38.1	<2E-16

^a^Genes coding for proteins identified by BlastX of significant marker sequences out of a previous genome wide association study (GWAS) by Wehner et al. [20]

### Relative expression of candidate genes

**Fig. 2:**
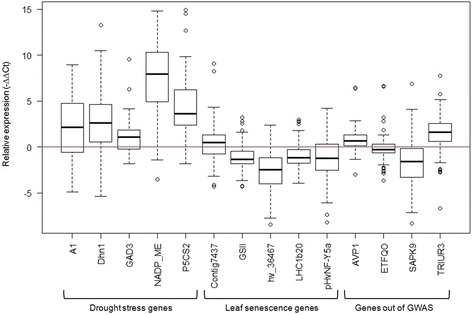
Expression profile for drought stress and leaf senescence genes. Relative Expression (-∆∆Ct) for the selected genes at 26 days after sowing (das) shown in box whisker plots including all 156 analysed barley genotypes

At the same developmental stage (BBCH 25) expression analyses were conducted for the whole set of 156 genotypes analysing 14 genes (Table 1). The relative expression (-∆∆Ct) ranges from −8.5 to 14.9 (Fig. 2, Additional file 1). In most genotypes all five drought stress related genes (A1, Dhn1, GAD3, NADP_ME and P5CS2) showed a higher expression under stress treatment relative to the control whereas for genes involved in leaf senescence opposite effects were detected for all genes (GSII, hv_36467, LHC1b20 and pHvNF-Y5α) except Contig7437. The genes out of the GWAS [20], i.e. AVP1 and TRIUR3 which are drought stress related genes, were up-regulated, whereas SAPK9 and ETFQO showed a lower expression relative to the control. In total, eight genes were up and six genes were down-regulated relative to the control but not all genotypes responded in the same way.

The mean quality score for all amplifications was 0.954. Because ∆Ct and ∆∆Ct values were not normally distributed (data not shown) further statistical analysis was done with logarithmic values (log_2_). Analysis of variance (ANOVA) revealed significant (*p* <0.001) effects for genotype and treatment for the 14 genes except Contig7437 (Table 2).

Highest significant correlations for differences in gene expression were identified within groups, i.e. within the group of drought stress genes, leaf senescence genes and genes out of GWAS (Table 3). The highest correlation was observed for the group of drought stress genes between relative expression of GAD3 and P5CS2 (r = 0.84), for the group of leaf senescence genes for GSII and pHvNF-Y5a (r = 0.64), and for the genes out of GWAS between AVP1 and TRIUR3 (r = 0.54). For no gene the differential expression was significantly correlated to the expression differences of all other genes, but ETFQO was correlated to all except Dhn1, and GAD3 and Contig7437 were correlated to all except GSII and AVP1, and SAPK9 and NADP_ME, respectively. Significant correlations were also detected between the relative SPAD values for change in leaf colour and all leaf senescence genes except hv_36467 with the highest coefficients of correlation for GSII (r = 0.24) and pHvNF-Y5a (r = 0.34). Moreover, significant correlations were observed for relative SPAD values to two genes out of GWAS (r = 0.16 for AVP1 and r = 0.15 for TRIUR3).

**Table 3:** Coefficients of correlation for relative expression of the selected genes and the relative SPAD values

		A1	Dhn1	GAD3	NADP_ME	P5CS2	Contig7437	GSII	hv_36467	LHC1b20	pHvNF-Y5α	AVP1	ETFQO	SAPK9	TRIUR3
	Rel. SPAD	0.09	0.02	−0.10	0.01	0	−0.16*	0.24**	−0.13	0.19*	0.34***	0.16*	0.09	−0.15	0.15*
Drought stress genes	A1		0.68***	0.68***	0.44***	0.76***	0.38***	0.15	0.10	−0.16	−0.12	0.14	0.18*	0.37**	−0.11
	Dhn1			0.73***	0.35**	0.72***	0.64***	0.08	0.26**	−0.17*	−0.11	0.12	0.15	0.30*	−0.18*
	GAD3				0.43***	0.84***	0.65***	0	0.17*	−0.31***	−0.28***	0.09	0.20*	0.34**	−0.34***
	NADP_ME					0.49***	0.15	0.29*	0.15	−0.01	0.10	0.27*	0.24*	0.22	0.25*
	P5CS2						0.50***	0.17*	0.13	−0.19*	−0.09	0.10	0.18*	0.40**	−0.18*
Leaf senescence genes	Contig7437							−0.17*	0.45***	−0.24**	−0.35***	0.18*	0.16*	0.21	−0.25**
	GSII								0.09	0.55***	0.64***	0.47***	0.53***	0.18	0.44***
	hv_36467									0.19*	−0.09	0.15	0.30***	0.03	0.01
	LHC1b20										0.49***	0.38***	0.39***	0.10	0.39*
	pHvNF-Y5α											0.42***	0.28***	−0.26*	0.41***
Genes out of GWAS^a^	AVP1												0.46***	0.22	0.54***
	ETFQO													0.17*	0.35*
	SAPK9														0.06

r is significant with *p <0.05, **p <0.01 and ***p <0.001

^a^Genes coding for proteins identified by BlastX of significant marker sequences out of a previous genome wide association study (GWAS) by Wehner et al. [20]

### Genome wide association study

Significant (*p* <0.001) marker gene expression associations were detected on all barley chromosomes except 4H with the highest number on chromosome 5H (8 single nucleotide polymorphisms, SNP) (Table 4). The largest transcriptional variance was explained by the marker SCRI_RS_181376 associated to the expression of ETFQO (R^2^ = 11.55 %) and the highest likelihood of odds (LOD) was observed for the marker SCRI_RS_161614 associated to the expression of TRIUR3 (LOD = 3.82) on barley chromosome 5H. Five SNP were significantly associated to the relative expression of the genes for drought stress, six to those for leaf senescence and seven to the genes out of the previous GWAS. Within the group of drought stress genes, expression differences of three genes (A1, GAD3 and P5CS2) and within the group of leaf senescence genes expression differences of four genes (Contig7437, GSII, hv_36467 and pHvNF-Y5α) were associated to markers. Out of these, three were located on chromosome 3H at 142.1 cM. This eQTL was detected for the relative expression of two drought stress genes (GAD3 and P5CS2) and one leaf senescence gene (Contig7437) which were also highly and significantly correlated (Table 3). Furthermore, an eQTL was observed for the relative expression of A1 on chromosome 5H at 149.9 cM associated to two markers. Associations for the relative expression of three genes (AVP1, ETFQO and TRIUR3) out of the four GWAS genes were detected on barley chromosomes 3H and 5H. For the expression of TRIUR3 three markers were found on 5H at 44.5 cM, and the expression of AVP1 was associated to a marker on chromosome 5H at 62.5 cM.

**Table 4:** Significant marker gene expression associations (*p* <0.001) with positions of eQTL

	Gene (log ∆∆Ct)	Marker^b^	Chr.^b^	Pos. in cM^b^	*F* value	*p* value	-log p (LOD)	R^2^ in %
Drought stress genes	A1	SCRI_RS_134358	5H	149.9	7.45	8.86E-04	3.05	9.5
	A1	SCRI_RS_165400	5H	150.1	7.45	8.86E-04	3.05	9.5
	GAD3	BOPA2_12_31177	1H	38.0	7.81	6.03E-04	3.22	8.9
	GAD3	BOPA1_4403-885	3H	142.1	12.09	6.67E-04	3.18	6.9
	P5CS2	BOPA1_4403-885	3H	142.1	11.31	9.84E-04	3.40	7.5
Leaf senescence genes	Contig7437	BOPA1_4403-885	3H	142.1	7.36	9.05E-04	3.01	7.1
	GSII	BOPA2_12_30065	7H	40.4	11.36	9.60E-04	3.04	9.5
	hv_36467	BOPA1_6547-1363	1H	111.8	8.11	4.58E-04	3.02	7.9
	hv_36467	BOPA2_12_31461	2H	131.9	13.14	4.00E-04	3.34	11.2
	pHvNF-Y5a	SCRI_RS_152393	6H	64.4	11.48	9.09E-04	3.04	7.8
	pHvNF-Y5a	SCRI_RS_194841	7H	81.5	12.91	4.49E-04	3.35	8.7
Genes out of GWAS^a^	AVP1	SCRI_RS_140294	5H	62.5	13.46	3.42E-04	3.47	9.1
	ETFQO	BOPA1_10126-999	3H	53.3	7.44	8.37E-04	3.08	10.1
	ETFQO	SCRI_RS_181376	5H	143.1	8.34	3.86E-04	3.41	11.5
	TRIUR3	BOPA1_4392-450	5H	44.5	7.64	7.07E-04	3.15	9.9
	TRIUR3	BOPA2_12_30717	5H	44.5	7.64	7.07E-04	3.15	9.9
	TRIUR3	SCRI_RS_41519	5H	44.5	7.64	7.07E-04	3.15	9.9
	TRIUR3	SCRI_RS_161614	5H	139.7	15.17	1.51E-04	3.82	9.8

^a^Genes coding for proteins identified by BlastX of significant marker sequences out of a previous genome wide association study (GWAS) by Wehner et al. [20]

^b^Marker positions are based on Comadran et al*.* [101]

The five SNP significantly associated to the relative expression of drought stress genes and the seven markers associated to genes out of GWAS all marked *cis* eQTL, while two *trans* eQTL were detected for P5CS2 and AVP1 (Table 5). In contrast, for the six markers significantly associated to leaf senescence genes only one *cis* eQTL was observed for pHvNF-Y5α. In summary, seven *trans* eQTL were detected and eight *cis* eQTL for which the Morex contigs showed a high identity to the gene analysed. Furthermore, *cis* eQTL explained a higher transcriptional variance (R^2^) than those in *trans* (Table 4 and Table 5).

**Table 5:** Positions of the selected genes based on the barley Morex-contigs and their mode of action

	Gene	POPSEQ^b,c^	Chr.^b^	cM^b^	Identity in %^c^	eQTL^d^
Drought stress genes	A1	morex_contig_38178	5H	156.9	76	*cis*
	GAD3	morex_contig_790741	1H	42.0	81	*cis*
	GAD3	morex_contig_135241	3H	147.0	75	*cis*
	P5CS2	morex_contig_2549060	3H	30.2	76	*trans*
Leaf senescence genes	Contig7437	morex_contig_47765	4H	54.3	94	*trans*
	GSII	morex_contig_274546	7H	70.8	92	*trans*
	hv_36467	morex_contig_138818	1H	132.4	91	*trans*
	hv_36467	morex_contig_458133	2H	58.0	81	*trans*
	pHvNF-Y5a	morex_contig_244610	6H	76.0	100	*trans*
	pHvNF-Y5a	morex_contig_60611	7H	70.8	95	*cis*
Genes out of GWAS^a^	AVP1	morex_contig_80803	5H	44.1	75	*trans*
	ETFQO	morex_contig_6218	3H	51.8	95	*cis*
	ETFQO	morex_contig_1570014	5H	152.4	100	*cis*
	TRIUR3	morex_contig_81592	5H	42.0	88	*cis*
	TRIUR3	morex_contig_160473	5H	129.9	71	*cis*

^a^Genes coding for proteins identified by BlastX of significant marker sequences out of a previous genome wide association study (GWAS) by Wehner et al. [20]

^b^Gene positions are based on POPSEQ map (ibsc 2012)

^c^Morex contigs and identity comes out Blastn of the gene sequences against the Morex genome (ibsc 2012)

^d^ *cis* eQTL coincide with the location of the underlying gene (position <10 cM), whereas *trans* eQTL are located in other regions of the genome Druka et al. [11]

## Discussion

### Drought stress and leaf senescence genes

As shown by the significantly decreased SPAD values at 27 days after sowing (das, BBCH 25), drought stress had an accelerating influence on natural leaf senescence in barley (Fig. 1 and Table 2). Furthermore, the drought stress answer in this juvenile stage was observed by differential expression of 14 genes induced by drought stress or leaf senescence (Table 1, Fig. 2).

A1 is a gene which is induced by ABA or abiotic stresses like drought, cold and heat [19, 52, 53]. In the present study expression under drought stress was higher than in the well watered treatment (Fig. 2). This was also shown by several studies first in barley [53] and other species including transgenics [54–57]. Dehydrins (Dhn) are well known to be expressed under dehydration stress [58]. For instance Dhn1 is described to be up-regulated under drought stress in barley [59, 60] which was also found in this study (Fig. 2). The glutamate decarboxylase gene (GAD3) is regulated by calcium and the protein encoded by this gene catalyzes the reaction of glutamate to γ-aminobutyric acid (GABA) [61, 62]. GABA may be involved in drought stress [63] by up-regulation of genes encoding a GABA receptor [29] which was also shown in the present study (Fig. 2). The NADP-dependent malic enzyme-like (NADP_ME) is involved in lignin biosynthesis, and regulates cytosolic pH through balancing the synthesis and degradation of malate [64]. As described in a drought stress study on barley, this effect is used for control of stomatal closure during the day under water-deficit conditions [29]. Comparable to the present study (Fig. 2) the gene for NADP_ME turned out to be higher expressed under drought stress [29]. The delta 1-pyrroline-5-carboxylate synthase 2 gene (P5CS2) is included in proline synthesis [65]. Content of proline is still controversially discussed as an indicator for drought tolerance [66], but it was shown in a previous study that the proline content increased under drought stress [20]. For approving its role, this gene was selected and showed up-regulation under drought stress (Fig. 2). Up-regulation under drought stress was also observed in tobacco [67] and transgenic rice [68].

The Contig7437 is a senescence associated gene (SAG) which is up-regulated under drought stress, as also shown by Guo et al*.* [29] in barley for drought stress during the reproductive stage. Other analysed SAGs are hv_36467 and pHvNF-Y5α, which were down-regulated in most genotypes under drought stress in our study (Fig. 2) whereas in literature reverse effects are described. The gene hv_36467 is a SAG12 like gene which is a senescence associated cystein protease and turned out to be up-regulated during natural leaf senescence in barley [69] and during dark induced senescence in tobacco [70]. In *Arabidopsis thaliana* the gene NFYA5 similar to pHvNF-Y5α was analysed by microarrays showing that the expression of this gene was induced by drought stress and ABA treatments [71], as well as under nitrogen stress [72]. Our data indicate a specific regulation of these two genes under different conditions. The protein encoded by the glutamine synthetase 2 (GSII) gene was found in photosynthetic tissues where its main role is the re-assimilation of photorespiratory ammonia [73, 74]. During senescence, the activity of GSII decreased representing down-regulation of associated genes in rice [73], barley and wheat [75] which was confirmed in the present study (Fig. 2). With chlorophyll degradation during leaf senescence the light harvesting complexes (LHC) of PSI and PSII remain stable, but synthesis rates of apoproteins of LHC decrease early in senescence [76]. In the present study LHC1b20 was down-regulated for most genotypes during drought stress induced leaf senescence in juvenile barley (Fig. 2) which was also shown in rice [77] and barley [78, 79] for natural leaf senescence in the generative stage.

In this study, all five selected drought stress genes were up-regulated under drought stress (Fig. 2) according to literature which demonstrates a clear drought stress answer and a good experimental setup for detecting and analysing drought stress response. In contrast, four out of the five selected genes for leaf senescence were down-regulated (Fig. 2) because a few of these genes are involved in photosynthesis and chloroplast development. Results for three of these genes (Contig7437, GSII and LHC1b20) were in accordance with results known from literature, while this was not the case for two of them (hv_36467 and pHvNF-Y5α). However, for all of these genes the adverse effect was detected for some genotypes (Fig. 2). Results revealed that drought stress in early developmental stages of barley leads to premature induced leaf senescence as already observed by physiological parameters [20] and by expression analysis of drought stress and leaf senescence related genes in this study.

Expression differences in three genes (GAD3, P5CS2 and Contig7437) were significantly associated to barley chromosome 3H at 142.1 cM (Table 4). At this position also quantitative trait loci (QTL) were found for drought stress [20, 80] as well as for leaf senescence [81]. These facts and the high correlation of these genes (Table 3) make this eQTL very interesting for marker assisted breeding in barley.

### Genes out of GWAS

To verify the QTL identified for drought stress and drought stress induced leaf senescence by Wehner et al*.* [20] an expression profile and eQTL analysis was conducted with genes coding for proteins identified within respective QTL. The genes ETFQO, SAPK9, TRIUR3 and AVP1 were differentially expressed (Fig. 2).

The protein encoded by the electron transfer flavoprotein-ubiquinone oxidoreductase gene (ETFQO) is located in the mitochondria where it accepts electrons from ETF, transfers them to ubiquinone and acts downstream in the degradation of chlorophyll during leaf senescence [82, 83]. Expression studies showed that ETFQO is up-regulated under darkness induced leaf senescence [83, 84] whereas in this study on drought stress induced leaf senescence no clear direction was observed (Fig. 2). A gene coding for a serine/threonine-protein kinase (SAPK9) was analysed which can be activated by hyperosmotic stress and ABA in rice [85]. In the present study SAPK9 was down-regulated in most genotypes (Fig. 2). Furthermore, the abscisic acid-inducible protein kinase gene (TRIUR3) which is also involved in dehydration stress response [86] was differentially expressed. Until now, no relative expression analysis has been conducted for this gene, but a huge amount of ABA inducible genes are up-regulated under drought stress in rice [87]. In the present study TRIUR3 was also up-regulated under drought stress (Fig. 2). The nucleotide pyrophosphatase/phosphodiesterase gene (AVP1) is a gene which is up-regulated under drought stress [88] which was confirmed in the current study (Fig. 2). Expression of this gene was also observed in transgenics showing a higher drought stress tolerance [89–92].

Three of these genes (SAPK9, TRIUR3 and AVP1) were located within the QTL on barley chromosome 5H at 45 cM [20]. Furthermore, expression differences of two of them (TRIUR3 and AVP1) were again associated to markers on chromosome 5H around 45 cM (Table 4) and this position was also validated in the Morex genome (Table 5). A high and significant correlation between the relative expression data of both genes as well as to the relative SPAD values (Table 3) promotes this finding. At the same position on chromosome 5H two markers which turned out to be significantly associated to SPAD and biomass yield under drought stress treatment were identified [20]. So, these results [20] and those of this study give hint that the two SNP markers, i.e. BOPA1_9766-787 and SCRI_RS_102075 may be used in marker based selection procedures in barley breeding programmes aiming at the improvement of drought stress tolerance.

For the understanding of complex mechanisms, such as the process of drought stress tolerance and drought stress induced leaf senescence as a basis for future breeding activities it is of prime importance to understand how and when regulatory genes are activated and where they are located in the barley genome. Results of this study contribute to elucidate the regulation of drought stress induced leaf senescence during early developmental stages in barley. The present genetical genomics approach helps to localize and understand transcriptional regulation and gene interaction, both from *cis*-acting elements and *trans*-acting factors (Table 5). When analysing the expression regulation of the barley genome, *cis* eQTL were found for the genes A1, GAD3, pHvNF-Y5α, ETFQO and TRIUR3. Markers which were significantly associated to *cis* eQTL explained up to 11.55 % of the transcriptional variance (Table 4 and Table 5). Therefore, most of the strongest eQTL acted in *cis* which was also observed in previous eQTL studies [8, 93, 94].

Factors that act in *trans* regulating the expression levels of the genes of interest were mainly found for the group of leaf senescence genes. Some of these genes are described as SAGs (Contig7437, hv_36467 and pHvNF-Y5α), because up to now little is known about their function. Results of the present study give hint that these SAGs are regulated in *trans.*

## Conclusion

With respect to the expression of genes involved in drought stress response and early leaf senescence genotypic differences exist in barley. Major eQTL for the expression of these genes are located on barley chromosome 3H and 5H. The eQTL on chromosome 5H coincides with the QTL for drought stress induced leaf senescence identified in a previous GWAS [43]. Respective markers, i.e. BOPA1_9766-787 and SCRI_RS_102075 may be used in future barley breeding programmes for improving tolerance to drought stress and early leaf senescence, respectively.

## Methods

### Plant material and phenotypic characterisation

Phenotyping, genotyping and QTL analysis were conducted as described in Wehner et al. [20] on a set of 156 winter barley genotypes consisting of 113 German winter barley cultivars (49 two-rowed and 64 six-rowed, [95]) and 43 accessions of the spanish barley core collection (SBCC) [96]. The same set of genotypes as well as the same experimental design was used for expression- and eQTL analysis in the present study. In brief, trials were conducted in greenhouses of the Julius Kühn-Institut in Groß Lüsewitz, Germany and drought stress was applied in a split plot design with three replications per genotype and treatment (control, drought stress). In each pot four plants were sown and all leaves were tied up, except the primary leaf per plant. Drought stress was induced by a termination of watering at the primary leaf stage (BBCH 10, according to Stauss [51]) seven days after sowing (das). From this time drought stress developed slowly till 20 das when the final drought stress level was reached. The drought stress variant was kept at 20 % of the maximal soil water capacity and the control variant at 70 % by weighing the pots resulting in a relative water content (36 das) ranging between 88.8 % and 91.5 % in the control variant and 80.9 % and 86.1 % in the drought stress treatment. The experimental setup and growth conditions for these pot experiment are described in detail as design B in Wehner et al. [20].

At 26 das (BBCH 25) leaf material for RNA extraction was sampled by harvesting one primary leaf per pot taking the middle part for further analyses. Mixed samples out of the three leaf pieces (circa 100 mg) per genotype and treatment (312 samples) each were immediately frozen in liquid nitrogen and stored at −80 °C. Furthermore, to get information on the influence of drought stress on leaf senescence leaf colour (SPAD, Konica Minolta Chlorophyll Meter SPAD-502 Plus, Osaka Japan) was measured 27 das on three primary leaves per pot at five positions each.

### RNA isolation and cDNA synthesis

The frozen primary leaves were homogenized with a tube pestle (Biozym) in liquid nitrogen. Total RNA from the primary leaves was isolated with the InviTrap Spin Plant RNA Mini Kit (STRATEC Molecular), using lysis solution RP and following the manufacturer’s instructions. After incubation for 15 min at room temperature, an additional incubation for 3 min at 55 °C was conducted to get a higher RNA yield. Total RNA yield was measured by Qubit fluorometric quantification (Life technologies) and concentration was adjusted to 50 ng. RNA was used for cDNA synthesis with the QuantiTect Reverse Transcription Kit (Qiagen) following the manufacturer’s instructions. cDNA was stored at −20 °C.

### Expression analysis using quantitative real-time PCR (qPCR)

A high throughput system (BioMark) was used for expression analysis in which four Fluidigm chips (96.96) were analysed for the 312 samples. Default space on these chips allows to analyse 48 genes in two technical replications. Out of these 48 analysed genes (23 genes involved in drought stress, 12 leaf senescence genes, 11 genes coding for proteins out of a previous GWAS [20] and two reference genes), 14 differentially expressed genes revealing clear differences between genotypes and showing a low number of missing values were selected for the present study. Five of these genes were involved in leaf senescence, five in drought stress response and four genes coding for proteins related to leaf senescence or drought stress out of the previous genome wide association study [20] were chosen. In addition, as a reference gene GAPDH was included (Table 1). To identify the gene for those proteins identified in the GWAS studies by Wehner et al. [20] the significant associated marker sequences were compared to the plant nucleotide collection by Blastn (Basic Local Alignment Search Tool, ncbi [www.ncbi.nlm.nih.gov] accessed June 2014) and the gene with the best hit was chosen for primer design.

Primers (Eurofins HPSF purified) were constructed using the primer designing tool of NCBI ([www.ncbi.nlm.nih.gov/tools/primer-blast] accessed June 2014) with a length of 20 bp, annealing temperature of 59 °C and product size of 100–200 bp (Table 1).

qPCR was performed using the high throughput platform BioMark HD System and the 96.96 Dynamic Array IFC (Fluidigm) following the manufacturer’s instructions. 5 μl Fluidigm sample premix consisted of 1.25 μl pre-amplified cDNA, 0.25 μl of 20x DNA binding dye sample loading reagent (Fluidigm), 2.5 μl of SsoFast EvaGreen Supermix with low ROX (BioRad) and 1 μl of RNase/DNase-free water. Each 5 μl assay premix consisted of 2 μl of 100 μM primers, 2.5 μl assay loading reagent (Fluidigm) and 0.5 μl RNase/DNase-free water. Thermal conditions for qPCR were: 95 °C for 60 s, 30 cycles of 96 °C for 5 s, 60 °C for 20 s plus melting curve analysis. Data were processed using BioMark Real-Time PCR Analysis Software 3.0.2 (Fluidigm). The quality threshold was set at the default setting of 0.65 and linear baseline correction and automatic cycle threshold method were used.

### Data analysis

The analysis software (Fluidigm Real- Time PCR Analysis Software) gave cycle threshold (Ct) values and calculated ∆Ct values, as well as a quality score for each amplification. Out of these ∆Ct values calculated out of the Ct value of the gene of interest minus the Ct value of the housekeeping gene (GAPDH) for each genotype, treatment and replication, the relative expression (∆∆Ct) was calculated out of the ∆Ct values for stress treatment minus the ∆Ct values for control treatment for each genotype and replication [97]. ∆∆Ct values without correction of PCR efficiency were used for calculation, because genes were tested and selected by their efficiency in preliminary experiments. A mean PCR efficiency (Quality Score of Fluidigm) was calculated for all amplifications.

Shapiro-Wilk test for normal distribution and analysis of variance (ANOVA) using a linear model were carried out using R 2.15.1 [98] to test effects of genotype (using ∆∆Ct values) and treatment (using ∆Ct values). Furthermore, coefficients of correlation (Spearman) were calculated in R between relative expression of the genes and the relative SPAD values [20, 99]. Moreover, for the SPAD values an ANOVA mixed linear model (MLM) was calculated (replication as random) in R to test effects of genotype, treatment and interaction of genotype and treatment. For relative expression as well as for the SPAD values box whisker plots were calculated in R.

### Expression quantitative trait loci (eQTL) analysis

For the 14 selected genes a genome wide association study (GWAS) for eQTL detection was conducted on the 156 genotypes applying a mixed linear model (MLM) using TASSEL 3.0 [100]. For this purpose a genetic map with 3,212 polymorphic SNP markers with minor allele frequencies larger than 5 % [101], a population structure calculated with STRUCTURE 2.3.4 [102] based on 51 simple sequence repeat (SSR) markers covering the whole genome, a kinship calculated with SPAGeDi 1.3d [103] based on 51 SSRs and the relative expression data (means for replications) were used. For comparability the methods were the same as used for GWAS in Wehner et al. [20]. All results with *p* values <0.001 (likelihood of odds, LOD = 3) were considered as significant marker gene expression associations.

To compare genomic positions of the eQTL with those of the analysed genes, sequences of the genes were compared against high confidential genes (CDS sequences) of the barley Morex genome by Blastn (Basic Local Alignment Search Tool, IPK Barley Blast server [http://webblast.ipk-gatersleben.de/barley/viroblast.php] accessed May 2015) and the Morex contig with the highest identity on the associated linkage group (chromosome) was chosen. With this information eQTL were divided in *cis* and *trans* eQTL. *cis* eQTL coincide with the location of the underlying gene (position <10 cM), whereas *trans* eQTL are located in other regions of the genome [11].

## Additional file

Additional file 1:
**Relative expression of the 14 genes with mean quality scores for each amplification.** ^a^SBCC: spanish barley core collection. ^b^GWAS: genome wide association study. (XLSX 111 kb)

## Abbreviations

∆∆Ct: relative expression
ABA: abscisic acid
Blast: Basic Local Alignment Search Tool
Ct: cycle threshold
das: days after sowing
e.g: for example
eQTL: expression quantitative trait locus/loci
GWAS: genome wide association study
i.e: id est
LEA: late embryogenesis abundant protein
LOD: likelihood of odds
MLM: mixed linear model
PCR: polymerase chain reaction
qPCR: quantitative real-time polymerase chain reaction
QTL: quantitative trait locus/loci
ROS: reactive oxygen species
SAG: senescence associated genes
SBCC: Spanish Barley Core Collection
SNP: single nucleotide polymorphism
SPAD: soil plant analysis development; measurement of chlorophyll content by colour
SSR: single sequence repeat
